# Artificial Intelligence–Powered Digital Health Platform and Wearable Devices Improve Outcomes for Older Adults in Assisted Living Communities: Pilot Intervention Study

**DOI:** 10.2196/19554

**Published:** 2020-09-10

**Authors:** Gerald Wilmink, Katherine Dupey, Schon Alkire, Jeffrey Grote, Gregory Zobel, Howard M Fillit, Satish Movva

**Affiliations:** 1 CarePredict Plantation, FL United States; 2 Lifewell Senior Living Corporation Houston, TX United States; 3 Department of Geriatric Medicine and Palliative Care Icahn School of Medicine Mount Sinai New York, NY United States; 4 Alzheimer’s Drug Discovery Foundation New York, NY United States

**Keywords:** health technology, artificial intelligence, AI, preventive, senior technology, assisted living, long-term services, long-term care providers

## Abstract

**Background:**

Wearables and artificial intelligence (AI)–powered digital health platforms that utilize machine learning algorithms can autonomously measure a senior’s change in activity and behavior and may be useful tools for proactive interventions that target modifiable risk factors.

**Objective:**

The goal of this study was to analyze how a wearable device and AI-powered digital health platform could provide improved health outcomes for older adults in assisted living communities.

**Methods:**

Data from 490 residents from six assisted living communities were analyzed retrospectively over 24 months. The intervention group (+CP) consisted of 3 communities that utilized CarePredict (n=256), and the control group (–CP) consisted of 3 communities (n=234) that did not utilize CarePredict. The following outcomes were measured and compared to baseline: hospitalization rate, fall rate, length of stay (LOS), and staff response time.

**Results:**

The residents of the +CP and –CP communities exhibit no statistical difference in age (*P*=.64), sex (*P*=.63), and staff service hours per resident (*P*=.94). The data show that the +CP communities exhibited a 39% lower hospitalization rate (*P*=.02), a 69% lower fall rate (*P*=.01), and a 67% greater length of stay (*P*=.03) than the –CP communities. The staff alert acknowledgment and reach resident times also improved in the +CP communities by 37% (*P*=.02) and 40% (*P*=.02), respectively.

**Conclusions:**

The AI-powered digital health platform provides the community staff with actionable information regarding each resident’s activities and behavior, which can be used to identify older adults that are at an increased risk for a health decline. Staff can use this data to intervene much earlier, protecting seniors from conditions that left untreated could result in hospitalization. In summary, the use of wearables and AI-powered digital health platform can contribute to improved health outcomes for seniors in assisted living communities. The accuracy of the system will be further validated in a larger trial.

## Introduction

Advances in public health and medical treatment over the past century have increased the average life expectancy in the United States by 30 years [1,2]. The number of people aged 65 years and older in the US is projected to more than double from 46 million today to 98 million by 2060 [3]. Individuals aged 85 years and older are the most rapidly growing age segment and have a growth rate that is four times that of the total population [4]. This age group is projected to triple from 6 million today to nearly 20 million by 2060, and this demographic shift is driving unprecedented needs for eldercare [5,6].

Older adults are disproportionally affected by chronic conditions, where 77% have at least two, and 65% have four or more chronic diseases [7-10]. Consequently, older adults tend to have the highest disability rate, the greatest need for long-term care services, and are more likely to be widowed and without someone to assist with activities of daily living [10,11]. Adults that suffer from chronic diseases that affect their mobility, independence, and ability to perform activities of daily living tend to require personal assistance in their home from either paid or unpaid caregivers, and if their needs are extensive may require relocation from their home to senior assisted living communities [5,10,12].

Assisted living is a long-term care option that combines housing, personal assistance with activities of daily living, and supportive specialized services and therapy. In the US, approximately 812,000 older adults live in nearly 29,000 assisted living communities [13-15]. Assisted living residents are, on average, 87 years of age, suffer from multiple chronic conditions, and nearly 75% require assistance with at least one activity of daily living [13-16]. Assisted living communities are beginning to utilize various types of technologies to maintain the health of their residents and increase their length of stay in the community [17]. Unplanned hospitalizations lead to decreased lengths of stay and are one of the primary causes of residents moving out of assisted living communities [18]. Hospitalizations are also a strong predictor of future nursing home admission and are associated with health declines, lower quality of life, and greater health care costs [18]. Falls are one of the leading causes for the hospitalization of residents in assisted living communities [19-24], and over the past decade, the fatality fall rate for adults age 85 and older has increased 41% [25,26]. Incident fall rates in assisted living communities range between 1.07 and 3.5 falls per person per year [23,27,28], and falling once doubles an older adult’s risk of falling again [29]. The fear of falling can also drive seniors to limit their activities, which can result in further physical decline, depression, and social isolation [30]. Fall detection technologies have improved in recent years; however, considering that 20-30% of falls are preventable [25,26], technologies are needed to predict and prevent falls [20,31-33].

Many caregivers in assisted living communities rely solely on their observational powers to detect health changes in older adults in their care. As the number of residents requiring more assistance is increasing in these communities, the number of available caregivers is decreasing dramatically. Technologies can be utilized to augment and force-multiply human observation and provide quality care. Such solutions can provide continuous observation, detecting changes in activity and behavior patterns that may be indicative of a change in health status—information that cannot be provided by intermittent human observation. Artificial intelligence (AI) can be used to bridge the caregiver-senior ratio gap and augment occasional human observation with continuous machine observation and deep learning neural nets to predict when interventions are needed.

A growing body of evidence demonstrates that sensor-laden wearables utilizing AI, and in particular machine-learning algorithms, can detect an individual’s daily activity and behavior [17,34-41]. In 1995, Celler et al developed the first telemonitoring system that could remotely monitor an older adult’s functional health status by continuously measuring their interactions over time [42]. The system measured a user’s mobility, sleep patterns, and utilization of cooking, washing, and toilet facilities to identify changes in functional health status [42]. In recent years, several monitoring technologies have been developed: radar sensing systems, passive infrared motion sensors, body-worn wearables, camera and video monitors, pressure sensors, and sound recognition [36,43-45]. Passive, ambient nonwearable based systems have found utility in home settings where one individual resides; however, such systems have difficulty accurately identifying a unique individual’s activity and behavior in a senior living community where many residents and staff members work and live [43]. The CarePredict AI-powered digital health platform, wearable device, and location system was developed for the autonomous, continuous, longitudinal measurement of activity and behavior patterns for multiple older adults and caregivers in a community setting [46-48]. The system measures a senior’s activity and behavior and can detect when such behavior is outside of their individual baseline. Caregiving staff can use the system to identify residents that may have an increased probability for a fall, depression, or urinary tract infection (UTI), and thereby give them time to provide a proactive intervention, protecting seniors from conditions that, left untreated, could result in hospitalization.

In this study, we tested whether the use of the CarePredict system could effectively improve the care provided in senior living communities. Specifically, we assessed the impact on hospitalization rate, fall rate, length of stay, and staff response time in each assisted living community.

## Methods

### Study Design and Population

**Figure 1:**
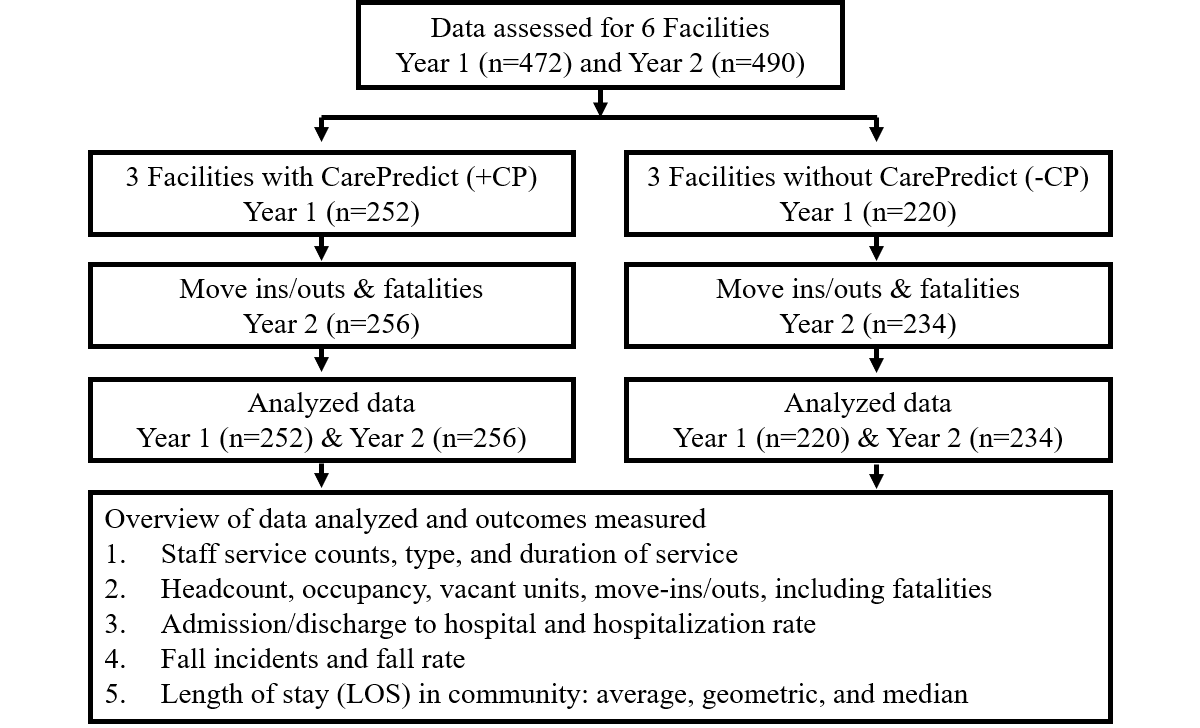
Workflow of data collection and analysis.

The study was designed to assess facility-level and resident-level outcomes for communities that utilized CarePredict’s AI-powered digital health platform, wearable device, and real-time location system. Retrospective analysis of anonymized resident data was collected from six assisted living communities in three states over 24 months. A study flow chart is provided in Figure 1. Data were analyzed for 472 residents in year 1 and 490 residents in year 2. The participants agreed to the collection of data presented in this publication by signing the terms and conditions for use, and data were anonymized for statistical analysis. Due to the nonexperimental, retrospective, de-identified, and anonymized study design, no ethical approval was needed.

### Data Collection

All de-identified data analyzed in this study were collected and reported by facility staff using each community’s online electronic healthcare software platform. The same software was used in all communities. CarePredict employees were provided access to the extracted anonymized data for scientific evaluation. No identifiable resident information has been or will be shared.

### Equipment and Process

**Figure 2:**
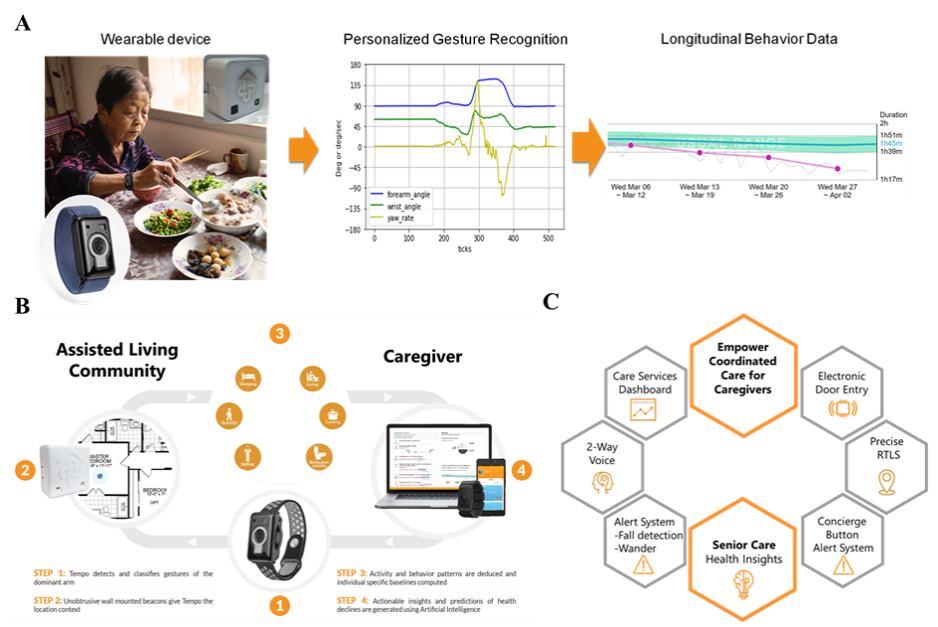
AI-powered digital health platform, wearable device, and room location system. A. Wearable device and sample representation of gesture recognition and activity detection. B. System architecture and overview of the data collection process. C. Summary of the product’s primary functions.

The CarePredict system consists of a wrist-worn wearable device, context beacons for room location, and a cloud-based AI-powered platform (Figure 2A-B). The wearable is worn on a user’s dominant arm, measures changes in their wrist kinematics, and autonomously quantifies gestures and activities of daily living such as eating, bathing, walking, bathroom visits, and sleep duration (Figure 2A-B). The wearable uses wireless communication to transfer data to the cloud over an encrypted connection and supports two-way audio that allows the resident to communicate to staff using mobile apps on iOS and Android devices. The wearable supports radio-frequency identification (RFID) protocols to allow integration with electronic door access systems enabling the resident to use their wearable for safe, secure, and convenient access to their apartment. The wearable measures 50 × 33 × 17.7 mm, weighs 40 g, and includes a six-axis accelerometer, a microprocessor, RFID, Bluetooth 4, Wi-Fi 802.11 b/g/n, and 1 Gb of onboard storage capable of storing 6 days of data. The wearable has a swappable battery, so the device does not need to be removed for charging. The battery is a 380mAH Li-ion 10.6g Polymer battery with 50 to 110 hours of battery life. The wearable has an operational temperature range of –20°C to +55°C, water-resistant to IP67, and the following certifications: FCC, CE, TELEC, RoHS, REACH, WEEE, Bluetooth.

The real-time location or context beacons enhance the accuracy of the wearable’s gesture recognition engine by bringing in room type data and permitting accurate room-level location tracking in an indoor setting [47]. Each beacon measures 52.1 × 52.1 × 28.0 mm, weighs 78 g, and uses Lithium CR123A batteries. The beacons utilize a proprietary line-of-sight technology to allow for real-time location monitoring on multi-floor levels with room-level accuracy and no bleed-throughs [46-48]. The CarePredict product is used in assisted living communities to provide five key functions (Figure 2C).

#### Actionable Insights

The system collects unique and rich data sets to train deep learning neural nets to surface crucial insights that correlate with an increased risk for a fall, UTI, or depression. A few correlates used in the system include the following: increased fall risk due to malnutrition, skipping meals, increased nightly get up count, reduced sleep duration, and decreased physical activity level; the increased probability for a UTI due to increased frequency or duration in bathroom visits, unusual toileting patterns, increased nighttime bathroom patterns, and reduced activity level; early signs of depression due to increased frequency of skipping meals, restless sleep, avoidance of bright lights and sunshine, and reduced physical activity levels. Further details on this system and established correlations are provided here [1,46-52]. All of these insights are generated without requiring self-reporting by the senior or need for another human observer. The power of this unique data set coupled with AI essentially provides a 24/7 net of continuous observation for the senior, giving caregivers insight into the evolving health of the senior so that proactive measures can be taken to avert a more severe health issue. A supplementary overview video on the system is provided in Multimedia Appendix 1 [53].

#### Real-Time Location System

Operators and staff both benefit from the use of an accurate real-time location system. First, staff can know the location of a resident who has pressed the button on their wearable to call for assistance, enabling the closest staff member to attend to a resident quickly. Second, historical insights allow the operators to assess previous shifts’ activities to improve staffing efficiencies. Such information may serve to facilitate improved response times, care coordination, and optimal workforce distribution. In addition, geofence alerts provide an added safety measure against wandering and elopement risks of residents with Alzheimer’s and dementia.

#### Documentation of Care Services

With a surge in acuity levels across senior living communities, providers need to have visibility into the amount of care required and provided to its residents. This solution allows caregivers to document at the point of care what services were rendered and a suite of reports that provide response time to alerts, time spent with residents on various direct care activities, and insights regarding quantity and the quality of care provided.

#### Two-Way Voice Communication

The wearable provides two-way voice communication that allows residents to communicate directly with the caregiving staff. Staff can prioritize alerts and respond appropriately. As a single communication platform for residents and staff, the wearable eliminates the need for multiple devices and provides tracking and reporting capabilities for staff efficiency.

#### Keyless Access Control

The wearable is integrated with passive RFID technology so it can be used for keyless door entry, providing convenience and safety to residents and staff and assuring consistent adherence of use.

### Outcome Measures

Facility staff at each community collected and reported the following data daily: occupancy, headcount, number of vacant units, unit move-ins and move-outs, staff service counts, duration, and type (such as dressing, bathing, grooming, transferring, and toileting), length of stay, fall incidents, emergency department and/or hospital admissions/discharges. Resident incident reports were utilized to document hospitalizations and fall incidents. The headcount, hospitalization, and fall incident numbers were used to compute both a hospitalization and fall rate. The hospitalization rate was defined as the number of hospitalization incidents per headcount in the facility, and the fall rate as the number of falls recorded per headcount per year. The average “baseline” rates for each community were measured from the first quarter of the study; the average “end of study” measurement was collected in the 8^th^ quarter of the study. The average rate of change between these periods was computed between these two periods. Staff response times were automatically measured in this study using the CarePredict system. Residents trigger an alert and call for staff assistance by depressing the button on their wearable, and a staff member acknowledges the alert using the CarePredict software. We analyzed both the duration of time the staff required to acknowledge an alert and then to reach the resident. The residents’ length of stay in each community was also measured. Length of stay is defined as the number of months a resident resides in a given community. The average, geometric, and median length of stay were analyzed, and detailed descriptions are provided in the supplementary materials section (Multimedia Appendix 2).

### Statistics

Descriptive statistics, including means, standard deviations, and distributions, were provided for all study variables and compared across groups (+CP vs. –CP communities). Study variables were compared to baseline measurements for each group. A two-sample, two-tailed, t-test was applied for metric variables to test for significant differences between groups. A *P* value <.05 was considered statistically significant.

### Compliance with Ethical Guidelines

Informed consent was obtained from the communities and participants included in the study.

## Results

### Resident Demographics

The resident demographic data (age, sex) and facility staff service time were assessed. The average resident ages were 87.3 years (SD 1.2 years) for the +CP communities and 88.1 years (SD 1.6 years) for the –CP communities (Table 1). The percent of female residents was 66.2% (SD 3.8) in the +CP and 69.2% (SD 8.2) in the –CP communities. The +CP and -CP communities exhibited no statistical difference in resident age (*P*=.64) and gender (*P*=.63). The average staff service time (hours per headcount per month) for each +CP and -CP community is shown in Table 2. The average staff service hours per resident per month were statistically similar for the +CP and –CP communities (*P*=.94).

**Table 1:** Resident age in the CarePredict and control assisted-living communities.

Age group	CarePredict (N=252), n (%)	Control (N=220), n (%)	*P* value
Below 75 years	12 (4.76)	21 (9.55)	.64
75-80 years	40 (15.87)	26 (11.82)	.47
81-85 years	74 (29.37)	55 (25.00)	.44
86-90 years	69 (27.38)	60 (27.27)	.62
Over 90 years	57 (22.62)	58 (26.36)	.72

**Table 2:** Average staff service time (hours) spent per headcount per month. There was no significant difference between groups (*P*=.94).

		Hours	
**CarePredict Community, mean (SD)**		76.7 (20.9)	
	1		81
	2		54
	3		95
**Control Community, mean (SD)**		77.6 (2.6)	
	1		59
	2		71
	3		103

### Outcome Measures

#### Hospitalization and Fall Rates

The hospitalization and fall rates for the six assisted living communities are provided inTable 3. The average baseline hospitalization rates for the +CP and –CP communities were 48.8% (SD 7.3) and 39.1% (SD 2.5), respectively. Compared to baseline, the average change in hospitalization rate decreased by 15.0% (SD 8.51) for the +CP communities and increased 34.5% (SD 7.24) for the –CP communities (*P*=.04). Thus, the hospitalization rate for the +CP communities was 39.8% lower than the –CP communities, 33.8% (SD 6.0) versus 73.6% (SD 18.1), respectively (*P*=.02). The average fall rate (number of total fall incidents per headcount per year) and change in fall rate compared to baseline was measured for both the +CP and –CP communities (Table 3). There was no significant difference between the groups’ initial baseline fall rates (*P*=.3). Compared to baseline, the fall rates for the +CP communities decreased 1.01 (SD 0.57), and the –CP communities increased 0.82 (SD 0.55). These changes are statistically significant (*P*=.05). The average fall rate for the +CP communities was 69% lower than for the –CP communities, 0.97 (SD 0.28), and 3.11 (SD 0.75), respectively. The normalized fall rate between groups was statistically significant (*P*=.01).

**Table 3:** Outcomes: hospitalization and fall rates for six assisted living communities.

Community	CarePredict (+/-)	Hospital incidents per headcount, N=490			Falls per headcount, N=490		
		Baseline, n (%)	Change from baseline, (%)	End of study, n, % (SD)	Baseline, n, fall rate	Change from baseline	End of study, n, fall rate (SD)
							
1	–	70 (42.0)	18.7	74, 60.7 (11.2)	70, 2.46	1.38	74, 3.84 (0.5)
2	–	70 (37.2)	57.1	78, 94.3 (12.7)	70, 2.31	0.81	78, 3.12 (0.8)
3	–	80 (38.1)	27.8	82, 65.9 (3.1)	80, 2.11	0.28	82, 2.39 (0.1)
4	+	80 (45.1)	–18.2	84, 26.9 (6.7)	80, 1.92	–0.65	84, 1.27 (0.8)
5	+	84 (57.2)	–19.4	84, 37.8 (18.9)	84, 2.40	–1.67	84, 0.73 (0.5)
6	+	88 (44.0)	–7.3	88, 36.7 (10.1)	88, 1.62	–0.72	88, 0.90 (0.7)
							
Mean (SD)	–	39.1 (2.5)	34.5 (7.24)	73.6 (18.1)	2.29 (0.18)	0.82 (0.55)	3.11 (0.75)
Mean (SD)	+	48.8 (7.3)	–15.0 (8.51)	33.8 (6.0)	1.98 (0.39)	–1.01 (0.57)	0.97 (0.28)
Delta		9.7	–49.5	–39.8	0.16	–1.83	–2.14
*P* value		.21	.04	.02	.30	.05	.01

#### Length of Stay in Assisted Living Communities

The median, geometric, and mean length of stay in the CarePredict and control communities are provided in Table 4. Length of stay was significantly greater in the CarePredict communities than in the control communities.

**Table 4:** Length of stay in CarePredict and control communities.

Community	CarePredict	Control	Difference in CarePredict vs control (%)	*P* value
Median length of stay (SD)	214 (38)	128 (8.7)	67	.03
Geometric mean length of stay (SD)	178 (46)	92 (8.6)	93	.04
Mean length of stay (SD)	268 (42.4)	192 (18)	40	.03

#### Staff Response Time

The average time to alert acknowledgment improved by 230 seconds (*P*=.03), and staff response time improved by 263 seconds (*P*=.02) (Table 5).

**Table 5:** Average acknowledgment and response times at baseline and the end of the study.

Response	Baseline, seconds, mean (SD)	End of study, seconds, mean (SD)	Improvement (%)	*P* value
Acknowledge alert	580 (42)	349.5 (82)	40	.03
Reach resident	763.5 (78)	500 (35)	37	.02

## Discussion

### Principal Findings

In this pilot study, we assessed whether the use of a wearable device and AI-powered digital health platform could provide improved health outcomes for older adults in an assisted living community. We found that the communities with CarePredict (+CP) exhibited a 40% lower hospitalization rate, 69% lower fall rate, and 67% greater length of residence stay compared to control communities (–CP). Overall, the use of CarePredict technology in assisted living communities appears to contribute to improved outcomes and shows promise as an effective tool to provide a higher quality of care.

There are several possible explanations for these findings. First, since both the residents and staff wear the CarePredict device, it functions as both an effective communication platform and resident alert system allowing for the coordination of prompt care. The system also provides robust staff performance metrics, which can be used to encourage continually improving staff response times to residents who need aid. The alert system prevents minor situations from escalating to emergent situations requiring hospitalization. Residents desire prompt, attentive care in assisted living communities, and the CarePredict system helped contribute to the facility staff acknowledging alerts 40% faster and reach residents in response to those alerts in 37% less time [54,55]. The CarePredict system appears to provide community staff with an increased awareness of residents’ needs and allows them to provide more prompt care, and thus may directly contribute to the improved outcomes observed.

Second, the system provides the community staff with detailed information regarding each resident’s activities and behavior. Changes in an adult’s activity and behavior are well-characterized to precede health declines; therefore, staff can use this information to quickly identify older adults that are at an increased probability for a health decline and intervene much earlier [49,50]. For example, such information can be used to identify and flag older adults that show the earliest sign of a urinary tract infection (UTI). In particular, the CarePredict system identifies a new or marked increase in urination urgency or frequency, both well-established indicators for a symptomatic UTI [49-51]. These older adults can then be assessed, and if diagnosed with a UTI, can be provided antibiotics to treat the UTI before it may result in hospitalization [51]. This increased visibility provided by the system may also have contributed to the observed lower hospitalization rates and increased LOS.

Fall rates also appear to be positively impacted by the use of the CarePredict solution. The data shows that +CP communities exhibited a 69% lower fall rate than the –CP communities. Fall rates are known to increase steadily with age [20], and the rates vary considerably for older people in different settings. Lower fall rates (0.3-1.6 per person per year) are typically reported in independent living communities with relatively healthy adults (age ≥65 years), whereas higher fall rates (0.6-4.05 per person per year, mean 1.7) are observed in assisted living, memory care, and long-term care institutions [24,56,57]. In a recent systematic review on falls, the mean rate of falls was found to vary between 1.07 falls per person per year for a low-risk population, and up to 3.5 falls per person per year for a high-risk population [23]. The CarePredict solution appears to contribute to an observed fall rate (0.97) that is lower than average incident fall rates reported in the literature [23,27].

By identifying older adults whose activity and behavior pattern indicates decreasing mobility, staff can take pre-emptive action to mitigate senior fall risk, UTIs, or other incidents that may have required hospitalization. Reducing hospital admissions also helps to maintain census, reduce resident turnover, and increase resident LOS in the community. The data shows that +CP communities exhibited a nearly 40% lower average hospitalization rate than the –CP communities (33.8% versus 73.6%). Several senior living outcomes studies by Zimmerman et al and Hedrick et al reported average annual hospitalization rates of 51% and 40%, respectively [29,58]. The +CP communities exhibited hospitalization rates that were 18% and 7% lower than the averages reported by Zimmerman and Hedrick et al, respectively [29,58].

### Limitations

There are several limitations to this pilot study. First, the study was conducted at six assisted living communities with less than 500 total residents. This study needs to be replicated and results confirmed using a larger sample size of individuals. Second, the –CP communities did not use an alert response technology system in this study, and thus staff response times could not be collected and analyzed for the –CP communities. As a result, the impact that the CarePredict technology had on staff response times was only measured and analyzed for the +CP communities. We, therefore, could not compare the staff response times between the +CP and –CP communities, and rather only measured the response times for the +CP communities at baseline and end of the study. Third, staff in the +CP communities used the CarePredict technology system for multiple purposes: to acknowledge and respond to resident alerts, to communicate to residents and other staff members, and to autonomously collect resident activity and behavior data. Since Carepredict served multiple functions, it is difficult to attribute which of these system capabilities and data sets directly contributed to the improved outcomes.

### Future Studies

To better understand the mechanisms by which these improvements were provided, in future studies, we plan to include a control group of communities that only utilize the CarePredict system for alerting and communication purposes. The added value provided by the predictive analytics feature will then be easier to assess and quantify directly. These results will also allow us to assess the impact that the proactive, actionable data generated by the CarePredict system will have on identifying and preventing high-risk residents from being hospitalized. Finally, although the six communities assessed in this study had comparable resident demographics (age, gender) and staff service hours per resident, other factors like residents’ hospital and fall history, and quality of care indicators may also have contributed to the differences observed in the measured outcomes.

### Conclusions

The leading cause of residents moving out of assisted living communities is unplanned hospitalization [18]. Hospitalizations are a strong predictor of nursing home admission and are associated with health and disability declines, lower quality of life, and greater health care costs [29,58]. The findings of this study highlight that the CarePredict AI-powered digital health platform, wearable device, and location system shows promise to support caregiving staff in identifying older adults that have an increased probability for a health decline, and thereby give staff time to provide a proactive intervention and thus reduce the number of hospitalizations. AI-powered platforms and wearable devices show promise as assistive tools for senior living organizations to deliver improved outcomes. In future studies, we plan to explore the variables and specific mechanisms by which this technology can directly contribute to each performance metric and outcome.

## Appendix

Multimedia Appendix 1 Overview video on AI-powered digital health platform and wearable.

Multimedia Appendix 2 Definitions and methods for calculating length of stay.

## Abbreviations

AI: artificial intelligence
+CP: community with CarePredict
–CP: community without CarePredict (control)
LOS: length of stay
RFID: radio-frequency identification
UTI: urinary tract infection
