# Supplementary material for: Artificial Intelligence–Powered Digital Health Platform and Wearable Devices Improve Outcomes for Older Adults in Assisted Living Communities: Pilot Intervention Study
Source: JMIR Aging. 2020 Sep 10;3(2):e19554. doi: 10.2196/19554 (PMC7516685; doi:10.2196/19554)

**Multimedia Appendix 2 – Methods for calculating length of stay**

The residents’ length of the stay in the community was also measured in this study. Length of stay indicates how many months an average resident resides in a given community. Three metrics for length of stay (LOS) are commonly computed and used to assess community care: average, geometric, and median LOS.

The average length of stay (LOS) is simply the arithmetic mean of the data, where d represents the resident days and N represents the total number of residents. The average LOS calculation is biased by the outliers and gives a misleading assessment.


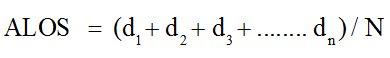


The geometric mean LOS is considered a more accurate or precise measurement as it represents the central value of an ensemble of points since it is not as sensitive to outliers. Given N residents the geometric mean is the Nth root of the product of the individual patient days. The geometric mean calculation is shown below:


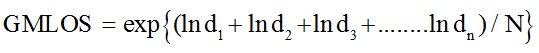


The third measurement is the median LOS. The median is the middle number in a sequence of numbers. Otherwise defined as the 2nd quartile or 50th percentile and best describes the central value of a distribution. The algorithm is based on the ALOS represented by h and the standard deviation of the LOS data. The median LOS is calculated using the following equation:


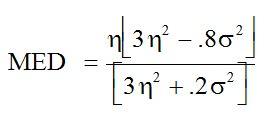

Supplement: Multimedia Appendix 2 [file aging_v3i2e19554_app2.docx]
